# Sjögren’s syndrome in a child presenting with cutaneous findings

**DOI:** 10.1186/1546-0096-12-S1-P290

**Published:** 2014-09-17

**Authors:** Ozge Basaran, Nilgun Cakar, Banu Celikel Acar, Nermin Uncu, Fatma Semsa Caycı, Gokce Gur, Aysel Taktak, Adem Yasin Koksoy, Esra Karakus

**Affiliations:** 1 Pediatric Nephrology and Rheumatology, Ankara Child Health Hematology Oncology Education and Research Hospital, Ankara, Turkey; 2 Pathology, Ankara Child Health Hematology Oncology Education and Research Hospital, Ankara, Turkey

## Introduction

Sjögren’s syndrome (SS) is a systemic autoimmune disease that presents with inflammation of lacrimal and salivary glands and accompanied by extraglanduler complications. The spectrum of disease extends from sicca symptoms to systemic involvement.In childhood, SS is an extremely rare autoimmune disorder (1).Here we present a girl with SS who was first presented with cutaneous lesions.

## Objectives

A 12-year-old girl was admitted to our hospital due to large erythematosus lesions on pretibial region that has been seen for three years. These lesions were started with hyperemic, tender and nodular forms then changed to a dark brown macular coloration and persists afterward. She did not suffer from dry mouth or eye. However, when the medical history was examined carefully, she pointed out that she had no teardrops. Erythrocyte sedimentation rate (ESR) was 43 mm/hour, C-reactive protein (CRP) was 0.43 mg/dl, antinuclear antibody test was 1/320 (+), anti-Ro and RF was positive and anti-La was negative. She had bilateral positive Schirmer test (2mm/2mm). Biopsy of the salivary gland revealed lymphocytic infiltration. With positive Schirmer test, positive anti-Ro antibody and ANA and supportive biopsy findings, the diagnosis of SS was established. She was started 200 mg/day hydroxycholoquine andsymptomatic therapy with eye drops.

## Methods

Case report.

## Results

Case report.

## Conclusion

Skin lesions may be the first symptoms of an underlying autoimmune disease. Although cutaneous involvement is highly seen in patients with SS, it is an uncommon disorder in pediatric population (2). Therefore, pediatricians should be aware of the possibility of a coexisting disease especially when the lesions are unusual.

## Disclosure of interest

None declared

